# Baseline levels of circulating galectin-1 associated with radiographic hand but not radiographic knee osteoarthritis at a two-year follow-up

**DOI:** 10.1016/j.ocarto.2024.100455

**Published:** 2024-03-01

**Authors:** M.L.E. Andersson, M. Zimmerman, E. Brogren, S. Bergman, L. Strindberg, E. Fryk, P.A. Jansson

**Affiliations:** a Spenshult Research and Development Center, Halmstad, Sweden; b Department of Environmental and Biosciences, School of Business, Innovation and Sustainability, Halmstad University, Halmstad, Sweden; c Department of Clinical Sciences, Section of Rheumatology, Lund University, Lund, Sweden; d Department of Orthopaedics, Helsingborg Hospital, Helsingborg, Sweden; e Department of Translational Medicine - Hand Surgery, Lund University, Lund, Sweden; f Department of Hand Surgery, Skåne University Hospital, Malmö, Sweden; g Primary Health Care, School of Public Health and Community Medicine, Institute of Medicine, Sahlgrenska Academy, University of Gothenburg, Gothenburg, Sweden; h Wallenberg Laboratory, Department of Molecular and Clinical Medicine, Institute of Medicine, Sahlgrenska Academy, University of Gothenburg, Gothenburg, Sweden

**Keywords:** Osteoarthritis, Knee, Hand, Galectin-1

## Abstract

**Objective:**

We tested the potential of circulating galectin-1, interleukin (IL)-1 beta, IL-6, and tumour necrosis factor alpha (TNF alpha) levels at baseline in individuals with knee pain as biomarkers for development of radiographic knee and/or hand osteoarthritis (OA).

**Design:**

This study comprised 212 individuals with knee pain from the Halland osteoarthritis cohort (HALLOA). Clinical characteristics and serum/plasma levels of galectin-1, IL-1 beta, IL-6, and TNF alpha were measured at baseline, and knee and hand radiographs were obtained at a two-year follow-up. The predictive value of circulating inflammatory markers and clinical variables at baseline was assessed using multinominal logistic regression for those who developed radiographic OA in knees only (n ​= ​25), in hands only (n ​= ​40), and in both knees and hands (n ​= ​43); the group who did not develop OA (n ​= ​104) was used as reference. Correlations were assessed using Spearman's correlation coefficients.

**Results:**

As expected, age was identified as a risk factor for having radiographic knee and/or hand OA at the two-year follow-up. Baseline circulating galectin-1 levels did not associate with developing radiographic knee OA but associated with developing radiographic hand OA (odds ratio (OR) for a 20% increased risk: 1.14, 95% confidence interval (CI) 1.01–1.29) and both radiographic knee and hand OA (OR for a 20% increased risk: 1.18, 95% CI 1.05–1.30). However, baseline IL-1 beta, IL-6, and TNF alpha did not associate with developing radiographic knee and/or hand OA.

**Conclusion:**

Non-age adjusted circulating galectin-1 is superior to IL-6, IL-1 beta, and TNF alpha in predicting radiographic hand but not knee OA.

## 1 Introduction

Although cartilage destruction and synovial inflammation are well-established features of osteoarthritis (OA), the pathophysiological mechanisms behind the disease are not fully understood. Over recent decades, the paradigm has shifted from regarding OA as merely a local joint disease towards that of a disease with systematic pathology [1]. Subclinical inflammation in cartilage, synovial fluid and bone may cause a leak of inflammatory markers from the affected joint into the blood circulation. However, to date, no circulating inflammation biomarker has been coupled with an increased risk of OA and whether the pathophysiology differs between OA in hands and knees is a matter of debate [1].

Galectin-1 is a carbohydrate-binding protein expressed in many tissues and acts as a regulator of inflammation. It associates not only with obesity and chronic diseases such as type 2 diabetes [2], but also with several rheumatic conditions, including OA [[3], [4], [5]]. Further, galectin-1 has been proposed to promote the degeneration of joint cartilage by activating proinflammatory genes, suggesting that it could be a feasible treatment target [6]. In OA, galectin-1 has been shown to predominantly accumulate in affected joint cartilage areas and to correlate with the degree of cartilage degradation [7].

The aim of this study was to determine the potential of circulating galectin-1, interleukin (IL)-1 beta, IL-6, and tumour necrosis factor (TNF) alpha levels at baseline in a cohort of individuals with knee pain as biomarkers for radiographic knee and/or hand OA identified at a two-year follow-up.

## 2 Method

### 2.1 Participants

The Halland osteoarthritis (HALLOA) cohort includes individuals with knee pain in the southwest of Sweden. Enrollment was between 2017 and 2019, and the inclusion criteria were: current knee pain with no known former radiographic knee OA, cruciate ligament rupture nor rheumatological disorder, and a preferred age between 30 and 65 years. The HALLOA cohort is registered at ClinicalTrials.gov, NCT04928170, and has previously been described in detail [8].

This cross-sectional study, with a two-year follow-up, included 212 individuals from the HALLOA cohort with knee and hand radiographs at a two-year follow-up (18% lacking knee and 25% lacking hand radiographs of the 306 originally included). There were no significant differences in age, fat mass or sex between drop-outs and those included. The included individuals had worse knee-related symptoms assessed with knee injury and osteoarthritis outcome score in all subscales compared to a healthy population [9] (data not shown). All participants gave their informed consent, and the study was approved by the Swedish Ethical Review Authority (Dnr: 2016/229, 2017/253, 2020-03866).

### 2.2 Clinical characterization at baseline

The participants were assessed by bioimpedance (InBody 770) to measure fat mass, and body mass index (BMI) was calculated. Circulating levels of HbA1c and C-reactive protein (CRP) (CRP ≥1.0 ​mg/L) were measured in accordance with current laboratory standards in Sweden. CRP <1.0 ​mg/L was further analysed with a sensitive CRP enzyme-linked immunosorbent assay (ELISA) method (Abnova, Taiwan). Insulin resistance was assessed by the triglyceride-glucose index (TyG), calculated by the formula Ln [fasting triglycerides (mg/dL) ​× ​fasting plasma glucose (mg/dL)/2]. Serum galectin-1 was measured using the Human Galectin-1 Quantikine ELISA Kit (Bio-techne, United Kingdom), coefficient of variation (CV) intra-assay 5.7% and inter-assay 7.5%, respectively. IL-1 beta (CV 4.4% and 10.7%), IL-6 (CV 3.6% and 4.9%), and TNF alpha (CV 2.2% and 6.7%) in serum were measured using human Quantikine, high-sensitive ELISA (Bio-techne, United Kingdom) for each cytokine.

### 2.3 Radiographic assessment at a two-year follow-up

Radiographic knee OA was defined, in accordance with Ahlbäck, as Ahlbäck grade I or more in at least one knee [10]. Ahlbäck classification does not include the patellofemoral joint, thus, radiographic knee OA in this study refers to weight-bearing joints. Radiographic hand OA was defined, based on the Kellgren-Lawrence (KL) classification [11], as having KL score of ≥2 in at least two groups (distal interphalangeal joints, proximal interphalangeal joints, and the first carpometacarpal joint) in at least one hand.

### 2.4 Statistical analysis

We compared baseline clinical characteristics for individuals without radiographic OA (no OA, n ​= ​104) versus those with radiographic OA in knees only (knee OA, n ​= ​25), in hands only (hand OA, n ​= ​40), and in both knees and hands (knee and hand OA, n ​= ​43) at a two-year follow-up. Most of the data were not normally distributed, so median and quartiles Q1-Q3 are presented. Kruskal-Wallis test was used to test the differences between groups. Missing data were not replaced. Associations between baseline variables and outcomes (radiographic knee OA and/or hand OA at the two-year follow-up) were calculated by multinominal logistic regression analyses, and if significant adjusted for age and fat mass. Not normally distributed data were log-transformed for near normal distribution in the regression model. Correlations at baseline with inflammatory biomarkers were assessed using Spearman's correlation coefficient. The significance tests were two-tailed, conducted at the 0.05 level of significance, and performed without Bonferroni corrections. Statistical analyses were performed using SPSS version 21.0 statistical software (IBM Corp., Armonk, NY, USA).

## 3 Results

### 3.1 Clinical characteristics at baseline

Of the 212 individuals, 104 (49%) had no radiographic OA, 25 (12%) had radiographic knee OA, 40 (19%) had radiographic hand OA, and 43 (20%) had both radiographic knee and hand OA at the two-year follow-up.

There were significant differences in baseline values for age (p ​< ​0.001), BMI (p ​= ​0.024) and HbA1c (p ​= ​0.006) and non-significant differences for baseline levels of circulating galectin-1 (p ​= ​0.05) between the groups defined by their radiographic OA outcome at the two-year follow-up. However, there were no differences between the groups for baseline levels of IL-1 beta (p ​= ​0.523), IL-6 (p ​= ​0.185) or TNF alpha (p ​= ​0.456) (Table 1).

**Table 1:** Comparisons at baseline between those without OA (no knee or hand OA) and those with knee OA, hand OA and both knee/hand OA at a two year-follow-up.

	No OA	Knee OA	Hand OA	Knee and hand OA	p-value
n	104	25	40	43	
Sex, female, n (%)	71 (68)	13 (52)	27 (68)	33 (77)	0.216
Age at inclusion	50 (42–55)	54 (50–58)	58 (52–60)	57 (55–60)	**<0.001**
BMI, kg/m^2^	25.1 (22.7–28.9)	28.8 (25.6–30.4)	25.2 (23.1–28.9)	26.9 (24.1–29.4)	**0.024**
Fat mass, kg	19.0 (15.4–28.1)	27.2 (19.7–32.9)	22.2 (15.6–28.2)	24.2 (18.4–31.0)	0.068
HbA1c, mmol/mol	36 (34–38)	37 (35–39)	38 (36–39)	38 (36–39)	**0.006**
TyG index	8.2 (7.9–8.5)	8.2 (7.9–8.8)	8.3 (8.1–8.6)	8.4 (8.1–8.9)	0.123
Galectin-1, ng/mL	27.0 (22.4–32.1)	27.8 (24.5–30.7)	28.9 (26.4–35.2)	29.3 (25.5–35.1)	**0.050**
CRP, mmol/L	1.0 (0.6–2.0)	1.2 (0.8–2.1)	1.1 (0.7–1.6)	1.4 (0.9–3.0)	0.126
IL-1 beta, pg/mL	0.05 (0.01–0.18)	0.11 (0.01–0.32)	0.06 (0.01–0.12)	0.07 (0.01–0.19)	0.523
IL-6, pg/mL	1.14 (0.77–1.79)	1.31 (0.90–1.91)	1.08 (0.86–1.60)	1.49 (0.93–2.32)	0.185
TNF alpha, pg/mL	1.09 (0.87–1.28)	1.24 (0.86–1.52)	1.08 (0.82–1.44)	1.14 (0.93–1.51)	0.456

BMI, body mass index; CRP, C-reactive protein; HbA1c, haemoglobin A1c; IL, interleukin; IQR, interquartile range; OA, Osteoarthritis; TNF, tumour necrosis factor; TyG, triglyceride-glucose. Median and interquartile range (Q1-Q3) presented, with the exception of sex. Kruskal-Wallis test used for comparisons between groups. The following data were missing: no OA group (fat mass n ​= ​2; HbA1c n ​= ​4; CRP n ​= ​1; TyG index n ​= ​2); knee OA (fat mass n ​= ​1); hand OA (HbA1c n ​= ​3); knee and hand OA (fat mass n ​= ​1; HbA1c n ​= ​1). The missing data were not replaced.

Bold numbers indicate statistically significant p-values.

### 3.2 Associations between clinical variables and radiographic OA

Older age and higher BMI at baseline, but not galectin-1, IL-1 beta, IL-6 or TNF alpha levels at baseline, were associated with radiographic knee OA at the two-year follow-up (Table 2).

**Table 2:** Multinominal logistic regression between baseline variables and OA in knees and/or hands at a two-year follow-up.

	Knee OA (n ​= ​25)			Hand OA (n ​= ​40)			Knee and hand OA (n ​= ​43)		
	OR	95% CI	p-value	OR	95% CI	p-value	OR	95% CI	p-value
**Covariates**									
Age, years	1.11	1.03–1.19	**0.004**	1.21	1.12–1.31	**<0.001**	1.24	1.14–1.34	**<0.001**
Fat mass, kg	1.03	0.99–1.08	0.117	0.99	0.96–1.03	0.728	1.02	0.99–1.06	0.259
**Mediators**									
HbA1c	1.14	0.82–1.59	0.424	1.28	0.97–1.70	0.08	1.14	0.86–1.50	0.344
TyG index	1.28	0.69–2.31	0.417	1.33	0.81–2.19	0.258	1.52	0.94–2.45	0.090
CRP	1.01	0.97–1.02	0.597	0.99	0.96–1.03	0.930	1.03	1.00–1.06	0.073
**Main effectors**									
Galectin-1	1.09	0.94–1.25	0.251	1.14	1.01–1.29	**0.029**	1.18	1.05–1.30	**0.007**
IL-1 beta	1.02	0.99–1.04	0.194	1.00	0.97–1.02	0.669	1.00	0.98–1.02	0.678
IL-6	1.03	0.98–1.05	0.255	0.99	0.95–1.04	0.762	1.04	1.00–1.08	0.068
TNF alpha	1.02	0.94–1.11	0.646	0.98	0.92–1.03	0.385	1.01	0.95–1.08	0.721
**Adjusted analyses**									
Galectin-1, adjusted for age	1.03	0.89–1.20	0.667	1.06	0.93–1.21	0.379	1.09	0.95–1.24	0.207
Galectin-1, adjusted for fat mass	1.04	0.90–1.21	0.598	1.16	1.02–1.32	**0.019**	1.17	1.03–1.33	**0.013**
Galectin-1, adjusted for age and fat mass	0.99	0.85–1.16	0.921	1.08	0.94–1.24	0.257	1.08	0.94–1.24	0.279

BMI, body mass index; CI, confidence interval; CRP, C-reactive protein; HbA1c, haemoglobin A1c; IL, interleukin; OA, Osteoarthritis; OR, odds ratio; TNF, tumour necrosis factor; TyG, triglyceride-glucose. For normally distributed data (age and fat mass), absolute ORs are presented. For data that are not normally distributed (mediators and main effectors), ORs are given for a relative increase of 20% in the risk of developing OA after two years.

Bold numbers indicate statistically significant p-values.

Older age and higher circulating levels of galectin-1 at baseline, but not IL-1 beta, IL-6 or TNF alpha levels at baseline, were associated with radiographic hand OA at the two-year follow-up (Table 2). The association between galectin-1 and radiographic hand OA remained after adjusting for fat mass but not after adjusting for age.

Older age and higher circulating levels of galectin-1 at baseline, but not IL-1 beta, IL-6 or TNF alpha levels at baseline, were associated with having both radiographic knee and hand OA at the two-year follow-up (Table 2). The association between galectin-1 and radiographic knee and hand OA remained after adjusting for fat mass, but not after adjusting for age.

### 3.3 Correlations between galectin-1 and other clinical variables at baseline

Given that galectin-1 was the only inflammatory biomarker associated with OA, we assessed correlations between galectin-1 and other clinical variables at baseline in the subgroups (Supplementary Table 1). In the no OA group, galectin-1 correlated with all clinical variables, except for HbA1c and TyG index. In the knee OA group, there were no significant correlations. In the hand OA group, galectin-1 correlated with age, TyG index, and the other assessed inflammatory biomarkers. In the knee and hand OA group, galectin-1 correlated with BMI, fat mass and TNF alpha (Supplementary Table 1). The other circulating inflammatory biomarkers at baseline also correlated with clinical variables in the different groups, but to a lesser extent than observed with galectin-1 (Supplementary Table 2).

## 4 Discussion

In a risk population with knee pain, non-age-adjusted circulating galectin-1 levels at baseline were associated with radiographic hand OA and with having both radiographic knee and hand OA, but not with radiographic knee OA, at a two-year follow-up. In contrast, circulating levels of IL-1 beta, IL-6 and TNF alpha did not differ between the groups at baseline and did not associate with having hand and/or knee OA. There were moderate correlations between galectin-1 and metabolic and inflammatory variables at baseline, especially in individuals who had radiographic hand OA at the two-year follow-up, whereas the other inflammatory biomarkers showed less salient associations. Thus, circulating non-age-adjusted galectin-1 appears to be a more robust inflammatory biomarker for predicting radiographic hand OA than circulating IL-6, IL-1 beta and TNF alpha, in individuals with knee pain.

Although hand OA and knee OA are known to share metabolic risk factors, such as obesity, dyslipidaemia and type 2 diabetes [12,13], our results suggest that the pathophysiological mechanisms of radiographic hand OA and knee OA may differ. In our study, galectin-1 was associated with hand but not knee OA at the two-year follow-up. We also showed that galectin-1 was associated with the insulin resistance marker TyG-index in the group with radiographic hand OA. Epidemiological studies have reported an association between galectin-1 and insulin resistance variables [2]. Preclinical work has also linked fatty acids to cartilage destruction [1]. Galectin-1 is involved in lipid storage in adipose cells [2] which suggests that further investigation of the interaction between galectin-1 and free fatty acids in cartilage is warranted.

In this study, galectin-1 was the only inflammatory biomarker associated with radiographic hand OA at a two-year follow-up. This supports our conjecture that galectin-1 might have a direct role in the pathophysiology of this disease. Higher age is a well-known risk factor in hand OA, and the association between galectin-1 and radiographic hand OA was lost after adjustment for age, suggesting that galectin-1 is a possible mediator of age-induced radiographic hand OA. Designated studies directly intervening with galectin-1 will provide answers to the question of whether there is any therapeutic relevance in targeting galectin-1 in radiographic hand OA.

Several mechanisms have been suggested for OA, e.g., “inflammageing”, with an accumulation of advanced glycosylation end products (AGEs) and increased inflammation [14]. HbA1c, a measure of glucose control, differed between groups at baseline, with higher levels in those individuals with radiographic OA in hands or knee and hands in the present study. Thus, increased glycosylation of proteins provides a potential mechanism for the low-grade inflammation seen in hand OA. In this study, radiographic knee OA was associated with age and obesity, which has also been shown in previous studies [1,15].

We found a correlation between baseline levels of galectin-1 and IL-1 beta, IL-6 and TNF alpha in the hand OA group and between galectin-1 and TNF alpha in the knee and hand OA group, which may indicate that galectin-1 has a role in the inflammatory process and is a manifestation of inflammation in hand OA. In the knee OA group, baseline galectin-1 levels did not correlate with any other clinical markers, suggesting that it is less likely that galectin-1 plays a significant role in knee OA. There were also correlations between galectin-1 and the other inflammatory biomarkers at baseline in those not fulfilling the criteria for radiographic knee OA or hand OA, i.e. the no OA group. Some of the individuals in the no OA group could have signs of OA but did not fulfil the criteria. Further studies are needed to better understand the reasons for this finding.

Limitations of the study were the relatively small size of the groups investigated, especially the knee OA group and no available data regarding radiographic OA of hands and knees at baseline or of other joints. Further, early radiographic features in hands and knees, evaluation of osteophytes or assessments of joint space narrowing was not evaluated and our definition of hand OA could have affected the result. The patellofemoral joint is not weight-bearing, and there could be different mechanisms compared to the tibiofemoral joint. The IL-1 beta levels were near the lower detection limit of the ELISA method, which could have had an impact on the findings. Finally, there was a difference in age between individuals who developed hand OA and those who developed knee OA. As galectin-1 is positively associated with age, the positive association of galectin-1 with radiographic hand but not knee OA might simply reflect the differences in ages between the groups. However, a strength of this study is that we predicted risk using established and novel circulating inflammatory biomarkers for knee OA in weight-bearing joints and hand OA in a uniquely phenotyped at-risk population with knee pain at baseline.

In conclusion, galectin-1 was the only non-age-adjusted inflammatory biomarker that could predict hand OA. Galectin-1 is an interesting target for further research in OA.

## Role of funding source

The HALLOA study was funded by the Swedish Rheumatism Association, grant numbers R-531621, R-635431, R-939824, and R-967899; targeted investment from the Swedish Rheumatism Association – Osteoarthritis from 2014 to 2019, and the Crafoord Foundation (MA), as well as the Kockska foundation, the Stig and Ragna Gorthon foundation and the regional agreement on medical training and clinical research between Region Skåne and Lund University (Yngre-ALF) (MZ). The study was supported by grants from the Swedish Research Council 2022-01011 (PAJ). The funders have not influenced the study design, collection, analysis, or interpretation of data, nor the writing of the manuscript or the decision to submit the manuscript for publication.

## Credit author statement

MA took part in the conception and design, collection and assembly of data, analysed the data, obtained the funding and drafted the manuscript; MZ and EB took part in the collection and assembly of the data, the design of the study, obtained funding and critically revised the article for intellectual content. SB took part in the analysis and interpretation of data and critically revised the article for intellectual content. EF took part in drafting the manuscript, interpretation of data, and critically revised the article for intellectual content. LS performed the ELISA analysis, took part in data interpretation, and critically revised the article for intellectual content. PAJ took part in the conception and design, the interpretation of data, obtained funding, took part in drafting the manuscript, and critically revised the article for intellectual content. All authors approved the final version to be submitted.

## Declaration of Generative AI and AI-assisted technologies in the writing process

The authors declare that they have not used any type of generative artificial intelligence for the writing of this manuscript.

## Declaration of competing interest

There are no conflicts of interest.
