# Supplementary material for: Baseline levels of circulating galectin-1 associated with radiographic hand but not radiographic knee osteoarthritis at a two-year follow-up
Source: Osteoarthr Cartil Open. 2024 Mar 1;6(2):100455. doi: 10.1016/j.ocarto.2024.100455 (PMC10926207; doi:10.1016/j.ocarto.2024.100455)
Supplement: Multimedia component 1 [file mmc1.docx]

|  | All  (n=212) |  | No OA  (n=104) | | Knee OA  (n=25) | | Hand OA  (n=40) | | Knee and hand OA  (n=43) | |
| --- | --- | --- | --- | --- | --- | --- | --- | --- | --- | --- |
|  | r_s_ | p-value | r_s_ | p-value | r_s_ | p-value | r_s_ | p-value | r_s_ | p-value |
| Age, years | 0.25 | **<0.001** | 0.25 | **0.012** | 0.23 | 0.267 | 0.15 | **0.012** | 0.12 | 0.446 |
| BMI, kg/m^2^ | 0.32 | **<0.001** | 0.37 | **<0.001** | 0.06 | 0.791 | 0.16 | 0.341 | 0.35 | **0.023** |
| Fat mass, kg | 0.28 | **<0.001** | 0.36 | **<0.001** | 0.04 | 0.869 | 0.12 | 0.454 | 0.31 | **0.043** |
| HbA1c, mmol/mol | 0.17 | **0.01** | 0.12 | 0.256 | 0.12 | 0.567 | 0.13 | 0.451 | 0.16 | 0.304 |
| TyG index | 0.21 | **0.003** | 0.18 | 0.072 | -0.06 | 0.764 | 0.45 | **0.004** | 0.07 | 0.646 |
| CRP, mg/L | 0.20 | **0.003** | 0.29 | **0.003** | -0.16 | 0.458 | 0.21 | 0.185 | 0.18 | 0.249 |
| IL-1 beta, pg/mL | 0.33 | **<0.001** | 0.38 | **<0.001** | 0.20 | 0.341 | 0.39 | **0.013** | 0.28 | 0.066 |
| IL-6, pg/mL | 0.31 | **<0.001** | 0.32 | **0.001** | 0.27 | 0.197 | 0.48 | **0.002** | 0.13 | 0.404 |
| TNF alpha, pg/mL | 0.44 | **<0.001** | 0.39 | **<0.001** | 0.22 | 0.288 | 0.62 | **<0.001** | 0.50 | **0.001** |

Supplementary Table 1. Correlations at baseline between galectin-1 and clinical data in individuals with no OA and those with knee OA, hand OA, and knee and hand OA at a two-year follow-up.

OA, osteoarthritis; BMI, body mass index; HbA1c, haemoglobin A1c; TyG, triglyceride-glucose; CRP, C-reactive protein; IL, interleukin; TNF, tumour necrosis factor. Spearman’s correlation coefficients (r_s_) and p-values are shown.
