# Supplementary material for: Baseline levels of circulating galectin-1 associated with radiographic hand but not radiographic knee osteoarthritis at a two-year follow-up
Source: Osteoarthr Cartil Open. 2024 Mar 1;6(2):100455. doi: 10.1016/j.ocarto.2024.100455 (PMC10926207; doi:10.1016/j.ocarto.2024.100455)
Supplement: Multimedia component 2 [file mmc2.docx]

| Age, years | 1 |  |  |  |  |  |  |  |  |  |
| --- | --- | --- | --- | --- | --- | --- | --- | --- | --- | --- |
| BMI, kg/m^2^ | 0.13; 0.03 | 1 |  |  |  |  |  |  |  |  |
| Fat mass, kg | 0.07; 0.34 | 0.87; <0.001 | 1 |  |  |  |  |  |  |  |
| HbA1c, mmol/mol | 0.40; <0.001 | 0.25; <0.001 | 0.25; <0.001 | 1 |  |  |  |  |  |  |
| TyG index | 0.18; 0.01 | 0.38, <0.001 | 0.31; <0.001 | 0.23; 0.001 | 1 |  |  |  |  |  |
| CRP, mg/L | 0.13; 0.06 | 0.52; <0.001 | 0.50; <0.001 | 0.17; 0.02 | 0.30; <0.001 | 1 |  |  |  |  |
| Galectin-1, ng/mL | 0.25, <0.001 | 0.32; <0.001 | 0.28; <0.001 | 0.17; 0.01 | 0.21; 0.003 | 0.20; 0.003 | 1 |  |  |  |
| IL-1 beta, pg/mL | 0.06; 0.36 | 0.08; 0.91 | 0.02; 0.97 | 0.02; 0.76 | 0.08; 0.22 | 0.08; 0.25 | 0.33; <0.001 | 1 |  |  |
| IL-6, pg/mL | 0.25; <0.001 | 0.36; <0.001 | 0.37; <0.001 | 0.26; <0.001 | 0.30; <0.001 | 0.51; <0.001 | 0.31; <0.001 | 0.11; 0.10 | 1 |  |
| TNF alpha, pg/mL | 0.15; 0.03 | 0.19; 0.0005 | 0.14; 0.04 | 0.19; 0.01 | 0.27; <0.001 | 0.12; 0.08 | 0.44; <0.001 | 0.26; <0.001 | 0.26; <0.001 | 1 |
|  | Age, years | BMI, kg/m^2^ | Fat mass, kg | HbA1c, mmol/mol | TyG index | CRP, mg/L | Galectin-1, ng/mL | IL-1 beta, pg/mL | IL-6, pg/mL | TNF alpha, pg/mL |

Supplementary Table 2A. Spearman correlation matrix between variables at baseline for all included (n=212, r_s_; p-level)

BMI, body mass index; CRP, C-reactive protein; HbA1c, haemoglobin A1c; IL, interleukin; TNF, tumour necrosis factor; TyG, triglyceride-glucose.

Supplementary Table 2B. Spearman correlation matrix between variables at baseline for the group without radiographic OA (n=104, r_s_; p-level)

| Age, years | 1 |  |  |  |  |  |  |  |  |  |
| --- | --- | --- | --- | --- | --- | --- | --- | --- | --- | --- |
| BMI, kg/m^2^ | 0.07; 0.47 | 1 |  |  |  |  |  |  |  |  |
| Fat mass, kg | 0.12; 0.22 | 0.88; <0.001 | 1 |  |  |  |  |  |  |  |
| HbA1c, mmol/mol | 0.40; <0.001 | 0.34; <0.001 | 0.28; 0.005 | 1 |  |  |  |  |  |  |
| TyG index | 0.20; 0.04 | 0.29; 0.003 | 0.19; 0.05 | 0.25; 0.01 | 1 |  |  |  |  |  |
| CRP, mg/L | 0.12; 0.21 | 0.56; <0.001 | 0.55; <0.001 | 0.17; 0.08 | 0.28; 0.005 | 1 |  |  |  |  |
| Galectin-1, ng/mL | 0.25; 0.01 | 0.36; <0.001 | 0.36; <0.001 | 0.12; 0.26 | 0.18; 0.07 | 0.28; 0.003 | 1 |  |  |  |
| IL-1 beta, pg/mL | 0.04; 0.68 | 0.04; 0.67 | 0.06; 0.52 | 0.02; 0,89 | 0.09; 0.38 | 0.02; 0.88 | 0.38; <0.001 | 1 |  |  |
| IL-6, pg/mL | 0.34; <0.001 | 0.38; <0.001 | 0.41; <0.001 | 0.32; 0.001 | 0.31; 0.001 | 0.53; <0.001 | 0.32; 0.001 | 0.06; 0.53 | 1 |  |
| TNF alpha, pg/mL | 0.06; 0.56 | 0.23; 0.02 | 0.08; 0.45 | 0.10; 0.35 | 0.20; 0.04 | 0.15; 0.13 | 0.39; <0.001 | 0.24; 0.02 | 0.24; 0.01 | 1 |
|  | Age, years | BMI, kg/m^2^ | Fat mass, kg | HbA1c, mmol/mol | TyG index | CRP, mg/L | Galectin-1, ng/mL | IL-1 beta, pg/mL | IL-6, pg/mL | TNF alpha, pg/mL |

BMI, body mass index; CRP, C-reactive protein; HbA1c, haemoglobin A1c; IL, interleukin; OA, Osteoarthritis; TNF, tumour necrosis factor; TyG, triglyceride-glucose.

| Age, years | 1 |  |  |  |  |  |  |  |  |  |
| --- | --- | --- | --- | --- | --- | --- | --- | --- | --- | --- |
| BMI, kg/m^2^ | -0.23; 0.27 | 1 |  |  |  |  |  |  |  |  |
| Fat mass, kg | -0.18; 0.39 | 0.90; <0.001 | 1 |  |  |  |  |  |  |  |
| HbA1c, mmol/mol | 0.45; 0.02 | -0.05; 0.81 | -0.05; 0.82 | 1 |  |  |  |  |  |  |
| TyG index | 0.26; 0.20 | 0.31; 0,13 | 0.37; 0.07 | 0.26; 0.21 | 1 |  |  |  |  |  |
| CRP, mg/L | -0.16; 0.45 | 0.49; 0.01 | 0.48; 0.02 | 0.18; 0.39 | 0.21; 0.32 | 1 |  |  |  |  |
| Galectin-1, ng/mL | 0.23; 0.27 | 0.06; 0.79 | 0.04; 0.87 | 0.12; 0.57 | -0.06; 0.76 | -0.16; 0.46 | 1 |  |  |  |
| IL-1 beta, pg/mL | -0.23; 0.27 | -0.19; 0.37 | -0.26; 0.23 | 0.06; 0.76 | -0.11; 0.61 | -0.04; 0.85 | 0.20; 0.34 | 1 |  |  |
| IL-6, pg/mL | 0.07; 0.73 | 0.14; 0.51 | 0.24; 0.25 | 0.31; 0.12 | 0.14; 0.50 | 0.46; 0.02 | 0.27; 0.20 | 0.16; 0.44 | 1 |  |
| TNF alpha, pg/mL | 0.38; 0.06 | 0.031; 0.88 | -0.07; 0.73 | 0.21; 0.32 | 0.03; 0.88 | -0.32; 0.11 | 0.22; 0.29 | 0.08; 0.69 | -0.01; 0.95 | 1 |
|  | Age, years | BMI, kg/m^2^ | Fat mass, kg | HbA1c, mmol/mol | TyG index | CRP, mg/L | Galectin-1, ng/mL | IL-1 beta, pg/mL | IL-6, pg/mL | TNF alpha, pg/mL |

Supplementary Table 2C. Spearman correlation matrix between variables at baseline for the group with radiographic knee OA (n=25), r_s_; p

BMI, body mass index; CRP, C-reactive protein; HbA1c, haemoglobin A1c; IL, interleukin; OA, Osteoarthritis; TNF, tumour necrosis factor; TyG, triglyceride-glucose.

| Age, years | 1 |  |  |  |  |  |  |  |  |  |
| --- | --- | --- | --- | --- | --- | --- | --- | --- | --- | --- |
| BMI, kg/m^2^ | -0.04; 0.83 | 1 |  |  |  |  |  |  |  |  |
| Fat mass, kg | -0.04; 0.79 | 0.85; <0.001 | 1 |  |  |  |  |  |  |  |
| HbA1c, mmol/mol | 0.47; 0.003 | 0.09; 0.60 | 0.25; 0.14 | 1 |  |  |  |  |  |  |
| TyG index | 0.08; 0.61 | 0.55; <0.001 | 0.34; 0.03 | 0.22; 0.19 | 1 |  |  |  |  |  |
| CRP, mg/L | 0.18; 0.25 | 0.30; 0.06 | 0.26; 0.10 | 0.05; 0.78 | 0.22; 0.17 | 1 |  |  |  |  |
| Galectin-1, ng/mL | 0.15; 0.35 | 0.16; 0.34 | 0.12; 0.45 | 0.13; 0.45 | 0.45; 0.04 | 0.21; 0.18 | 1 |  |  |  |
| IL-1 beta, pg/mL | 0.03; 0.86 | -0.24; 0.14 | -0.29; 0.07 | -0.29; 0.08 | 0.06; 0.71 | 0.17; 0.30 | 0.39; 0.01 | 1 |  |  |
| IL-6, pg/mL | 0.27; 0.10 | 0.23; 0.16 | 0.30; 0.06 | 0.07; 0.68 | 0.30; 0.06 | 0.37; 0.02 | 0.48; 0.002 | 0.02; 0.92 | 1 |  |
| TNF alpha, pg/mL | 0.18; 0.27 | 0.03; 0.85 | 0.10; 0.54 | 0.11; 0.52 | 0.14; 0.40 | 0.12; 0.48 | 0.62; <0.001 | 0.15; 0.37 | 0.15; 0.37 | 1 |
|  | Age, years | BMI, kg/m^2^ | Fat mass, kg | HbA1c, mmol/mol | TyG index | CRP, mg/L | Galectin-1, ng/mL | IL-1 beta, pg/mL | IL-6, pg/mL | TNF alpha, pg/mL |

Supplementary Table 2D. Spearman correlation matrix between variables at baseline for the group with hand OA (n=40, r_s_; p-level)

BMI, body mass index; CRP, C-reactive protein; HbA1c, haemoglobin A1c; IL, interleukin; OA, Osteoarthritis; TNF, tumour necrosis factor; TyG, triglyceride-glucose.

| Age, years | 1 |  |  |  |  |  |  |  |  |  |
| --- | --- | --- | --- | --- | --- | --- | --- | --- | --- | --- |
| BMI, kg/m^2^ | -0.12; 0.09 | 1 |  |  |  |  |  |  |  |  |
| Fat mass, kg | 0.09; 0.59 | 0.80; <0.001 | 1 |  |  |  |  |  |  |  |
| HbA1c, mmol/mol | 0.05; 0.74 | 0.12; 0.42 | 0.18; 0.27 | 1 |  |  |  |  |  |  |
| TyG index | -0.12; 0.44 | 0.36; 0.02 | 0.36; 0.02 | -0.10; 0.53 | 1 |  |  |  |  |  |
| CRP, mg/L | 0.08; 0.60 | 0.43; 0.004 | 0.38; 0.01 | 0.12; 0.44 | 0.31; 0.04 | 1 |  |  |  |  |
| Galectin-1, ng/mL | 0.12; 0.45 | 0.35; 0.02 | 0.31; 0.04 | 0.16; 0.30 | 0.07; 0.65 | 0.18; 0.25 | 1 |  |  |  |
| IL-1 beta, pg/mL | 0.28; 0.07 | 0.16; 0.32 | 0.36; 0.02 | 0.15; 0.35 | 0.24; 0.12 | 0.27; 0.08 | 0.28; 0.07 | 1 |  |  |
| IL-6, pg/mL | 0.03; 0.83 | 0.40; 0.008 | 0.32; 0.04 | 0.15; 0.34 | 0.18; 0.25 | 0.59; <0.001 | 0.13; 0.40 | 0.22; 0.16 | 1 |  |
| TNF alpha, pg/mL | 0.27; 0.08 | 0.19; 0.23 | 0.31; 0.04 | 0.22; 0.16 | 0.24; 0.12 | 0.28; 0.07 | 0.50; 0.001 | 0.51; <0.001 | 0.38; 0.01 | 1 |
|  | Age, years | BMI, kg/m^2^ | Fat mass, kg | HbA1c, mmol/mol | TyG index | CRP, mg/L | Galectin-1, ng/mL | IL-1 beta, pg/mL | IL-6, pg/mL | TNF alpha, pg/mL |

Supplementary Table 2E. Spearman correlation matrix between variables at baseline for the group with both hand and knee OA (n=43, r_s_; p-level)

BMI, body mass index; CRP, C-reactive protein; HbA1c, haemoglobin A1c; IL, interleukin; OA, Osteoarthritis; TNF, tumour necrosis factor; TyG, triglyceride-glucose
